# Clozapine Use Presenting with Pseudopheochromocytoma in a Schizophrenic Patient: A Case Report

**DOI:** 10.1155/2013/194927

**Published:** 2013-01-14

**Authors:** Jaskanwal Sara, Matt Jenkins, Tanveer Chohan, Karan Jolly, Lisa Shepherd, Nirav Y. Gandhi, Jayadave Shakher

**Affiliations:** ^1^University Hospitals Coventry and Warwickshire NHS Trust, Coventry CV2 2DX, UK; ^2^Waikato General Hospital, Hamilton 3240, New Zealand; ^3^University Hospitals Birmingham NHS Foundation Trust, Birmingham B15 2TH, UK; ^4^Walsall Healthcare NHS Trust, Walsall WS2 9PS, UK; ^5^Diabetes and Endocrinology, Birmingham Heartlands Hospital, Heart of England Foundation Trust, Birmingham B9 5SS, UK; ^6^College of Medical and Dental Sciences, University of Birmingham, Birmingham B15 2TT, UK

## Abstract

*Introduction*. There have been six previous cases that reported pseudopheochromocytoma in patients taking clozapine. Our case showed the direct link of clozapine to serum levels of certain markers. *Case*. This is a case of a 49-year-old obese Caucasian female who was referred to endocrinology for investigation of Cushing's syndrome, based on raised blood pressure and Cushingoid facies. The patient had underlying schizophrenia and was stable on clozapine. Her blood pressure was 150/99 mmHg on bendroflumethiazide and candesartan. We measured her 24-hour urinary-free cortisol, which was normal but 24-hour urinary-free noradrenaline was elevated at 835 nmol (76–561) with normal adrenaline 36 nmol (7–82) and dopamine 2679 nmol (366–2879), as the patient had history of palpitations and sweating. Two sets of 24-hour urinary-free cortisol measurements were normal and serum cortisol suppressed to <50 nmol/l after a 1 mg overnight dexamethasone. Two further 24-hour urinary-free catecholamines showed a raised level of noradrenaline. MRI demonstrated normal adrenals and MIBG scan did not show any abnormal uptake at adrenal glands. *Conclusion*. Pseudopheochromocytoma has been reported in patients taking clozapine. A number of different mechanisms for raised plasma noradrenaline levels with clozapine have been postulated. The above case highlights an unusual but known side effect of clozapine.

## 1. Introduction

Phaeochromocytoma is a very rare endocrine condition. The symptoms of phaeochromocytoma are varied and mimicked by many drugs and medical and surgical conditions. The common initial screening test is free urinary catecholamines and more specifically urinary metanephrines. Drugs can cause false positive elevation of plasma and urinary catecholamines or metanephrines.

We described a case of a 49-year-old lady with hypertension and paranoid schizophrenia who was treated with clozapine with false elevation of urinary-free catecholamines and metanephrines. This case illustrates difficulty in distinguishing a true case of phaeochromocytoma from a pseudopheochromocytoma especially in a symptomatic patient who is dependent on antipsychotic medications, which can cause false positive elevation of urinary-free catecholamines and metanephrines.

## 2. Case Report

A 49-year-old obese Caucasian female presented to the endocrine clinic following a GP referral for the investigation of a possible diagnosis of Cushing's disease due to a raised blood pressure and Cushingoid facies. The patient had underlying long-term schizophrenia and was stable on 100 mg of clozapine. Her blood pressure control was suboptimal despite being on bendroflumethiazide and candesartan.

Clinical examination demonstrated a Cushingoid habitus characterised by typical facies, a supraclavicular fat pad, and central adiposity. Her blood pressure was 150/99 mmHg and she appeared flushed. Examination of the remainder of her cardiovascular system was unremarkable and her visual fields and fundi were also normal.

Her 24-hour urinary-free cortisol was normal but 24-hour urinary-free noradrenaline was elevated at 835 nmol (76–561) with normal levels of adrenaline 36 nmol (7–82) and dopamine 2679 nmol (366–2879), which were requested by the GP in light of the patient's history of palpitations and sweating. Two sets of 24-hour urinary-free cortisol measurements were normal and serum cortisol suppressed to <50 nmol/l after a 1 mg overnight dexamethasone dose. Two further 24-hour urinary-free catecholamines showed raised levels of noradrenaline. Urea and electrolytes, liver function tests, and calcium were all normal.

An MRI demonstrated normal adrenals and a meta-iodobenzylguanidine (miBG) scan did not show any abnormal uptake in the adrenal glands. Based on these investigations, she was diagnosed with pesudopheochromocytoma due to clozapine, on which she was maintained at that time. She was lost to follow up and presented two years later at the hypertension clinic when she was on a different antipsychotic medication, zuclopenthixol. The repeat 24-hour urinary-free catecholamine as well as urinary normetadrenaline and metadrenaline were in the normal range. Urinary-free levels were as follows: noradrenaline 332 nmol, adrenaline 12 nmol, normetadrenaline 3.48 nmol (0.00–4.90), and metadrenaline 0.33 nmol (0.00–2.00).

## 3. Discussion

Using the keywords “hypertension,” “clozapine,” and “phaeochromocytoma” individually or in varying combinations, we searched a number of online databases for previous cases of pseudopheochromocytoma secondary to clozapine therapy. In all, six previous cases [1–3] were found comprising of five males and one female with ages ranging 27–44 years. The duration of clozapine therapy varied from two months to eighteen months.

Prior to initiating clozapine therapy, systolic blood pressure ranged 110–130 and diastolic blood pressure ranged 70–90. During therapy with clozapine, systolic blood pressure ranged 143–180 and diastolic blood pressure ranged 106–120. Furthermore, the heart rate during the period of elevated blood pressure ranged 100–130 and all six patients had elevated urinary catecholamines.

Clozapine was stopped in four out of six of the patients. In each case, the blood pressure and the 24-hour urinary catecholamine normalised after clozapine was stopped. Two of the patients continued with clozapine, one of whose blood pressure settled spontaneously.

Clozapine is a tricyclic dibenzodiazepine with complex pharmacological actions that include an affinity for a wide variety of receptors including alpha adrenergic, serotonergic, and muscarinic [4].

A number of studies have reported increased plasma noradrenaline levels in patients on clozapine treatment [5–8]. This would be reflected in elevated free urinary catecholamine levels [9], used as the investigation of choice in this case. A number of different mechanisms for raised plasma noradrenaline levels with clozapine have been postulated. These include that clozapine inhibits the synaptic reuptake of noradrenaline [7] and that clozapine blocks alpha-2-adrenoreceptors or the combination of the two [6, 7]. However, the findings of Elman et al. did not support the theory of decreased synaptic reuptake [8]. Instead, they suggested that clozapine's action might have a role in increasing the fusion of noradrenaline storage vesicles within the nerve axon. Hypertension has been described in as many as 4% of patients taking clozapine [6] which may be accounted for by the blockage of alpha-2-adrenreceptors [1, 2, 7]. Raised levels of noradrenaline may then spill over from the central nervous system into the plasma, which in turn will be present in urine, giving rise to the clinical features and biochemical abnormalities highlighted in our and previous cases.

There are a number of drugs besides clozapine that have been known to cause elevations in plasma and urinary catecholamines. It is therefore possible that the raised plasma noradrenaline levels identified in the cases found in our literature search may in fact be false positives for clozapine. Four patients were concurrently on antidepression medication, three patients were treated with antihypertensive medication, and two patients were also on sulpiride treatment, all of which may be implicated in raising plasma noradrenaline levels. Furthermore, sulpiride is known to block alpha-2-adrenorecepetors and an acute hypertensive episode has been described with its use [10]. This may have been responsible for, or at least contributed to, the clinical features of two of the reported cases by Krentz et al. [2] that the author himself mentioned.

Nevertheless, Breier et al. [7] compared clozapine to haloperidol and fluphenazine and found that clozapine patients had plasma noradrenaline levels almost five times that greater than those in haloperidol and fluphenazine. Similarly, Elman et al. [8] found that patients treated with clozapine had three times the plasma noradrenaline levels than those treated with fluphenazine or placebo. It appears as if elevation of circulating noradrenaline levels is a normal part of clozapine's action and may well occur to a greater degree than that found in other culprit drugs.

## 4. Conclusion

In conclusion, our patient developed pseudopheochromocytoma secondary to the initiation of treatment with clozapine, which subsided on cessation of the drug. Clozapine causes an elevation of plasma noradrenaline through any one of a number of possible mechanisms working individually or in concert. Though other drugs, including other antipsychotics, may also raise plasma noradrenaline levels, it appears as if clozapine does so to a greater extent. Hypertension and tachycardia also seem to be associated with clozapine therapy [11], and it seems likely that the pseudopheochromocytoma may result either directly from the elevated levels of plasma noradrenaline or may share a common causative mechanism through alpha-2-adrenreceptor blockade.

As hypertension has a potentially dangerous side effect with serious complications such as cardiac failure and other end-organ damage, the use of the drug must be considered carefully with the benefits outweighing any risks. Where clozapine is used, reports in the literature suggest that any resultant hypertension may be controlled with the use of appropriate medication.

This case was able to demonstrate the direct link of clozapine to raise urinary noradrenaline and normetadrenaline levels as these normalised after discontinuation of clozapine.
